# Systematic bibliometric and visualized analysis of research hotspots and trends in attention-deficit hyperactivity disorder neuroimaging

**DOI:** 10.3389/fnins.2023.1098526

**Published:** 2023-03-28

**Authors:** Haiyin Deng, Zhenming Huang, Zhaoying Li, Lei Cao, Youze He, Ning Sun, Yi Zeng, Jingsong Wu

**Affiliations:** ^1^Department of Rehabilitation Medicine, Fujian University of Traditional Chinese Medicine, Fuzhou, China; ^2^Rehabilitation Medicine Center and Institute of Rehabilitation Medicine, West China Hospital, Sichuan University, Chengdu, China; ^3^Innovation and Transformation Center, Fujian University of Traditional Chinese Medicine, Fuzhou, China

**Keywords:** attention-deficit hyperactivity disorder, neuroimaging, bibliometric, web of science, visualization

## Abstract

**Introduction:**

This study focused on the research hotspots and development trends of the neuroimaging of attention deficit hyperactivity disorder (ADHD) in the past thirty years.

**Methods:**

The Web of Science database was searched for articles about ADHD neuroimaging from January 1992 to September 2022. CiteSpace was used to analyze the co-occurrence of keywords in literature, partnerships between authors, institutions, and countries, the sudden occurrence of keywords, clustering of keywords over time, and analysis of references, cited authors, and cited journals.

**Results:**

2,621 articles were included. More and more articles have been published every year in the last years. These articles mainly come from 435 institutions and 65 countries/regions led by the United States. King's College London had the highest number of publications. The study identified 634 authors, among which Buitelaar, J. K. published the largest number of articles and Castellanos, F. X. was co-cited most often. The most productive and cited journal was Biological psychiatry. In recent years, burst keywords were resting-state fMRI, machine learning, functional connectivity, and networks. And a timeline chart of the cluster of keywords showed that “children” had the longest time span.

**Conclusions:**

Increased attention has been paid to ADHD neuroimaging. This work might assist researchers to identify new insight on potential collaborators and cooperative institutions, hot topics, and research directions.

## Introduction

Attention-deficit/hyperactivity disorder (ADHD) is the most prevalent neurodevelopmental disorder among children. The worldwide prevalence has been estimated between 2 and 7%, with an average of approximately 5% (Sayal et al., 2018). ADHD is manifested as poor sustained attention, impaired impulse control, and high hyperactivity (American Psychiatric Association, 2013). In addition, ADHD occurs throughout the patient's life cycle, sourcing from early childhood and extending to adulthood (Campbell et al., 2014; Franke et al., 2018).

A growing number of neuroimaging literature has started to explore the neural substrates and neuroimaging markers associated with ADHD. Some neuroimaging studies reported neurochemical, structural, and functional dysfunctions in the brain of patients with ADHD (Albajara Sáenz et al., 2019). Neuroimaging techniques are divided into two categories: structural and functional neuroimaging. Structural magnetic resonance imaging (sMRI) and diffusion tensor imaging (DTI) have been widely used in ADHD studies. Previous sMRI studies found that patients with ADHD had a decrease in overall brain volume (Narr et al., 2009; Nakao et al., 2011; Hoogman et al., 2017), as well as cortical thickness reductions, particularly in prefrontal, parietal, temporo-parietal, and occipital regions (Shaw et al., 2007; Wolosin et al., 2009; Almeida et al., 2010; Silk et al., 2016; Ambrosino et al., 2017). DTI study found an atypical junction of the corpus callosum in patients with ADHD (Aoki et al., 2018; Fuelscher et al., 2021).

Functional neuroimaging studies, including those employing functional MRI (fMRI), functional near-infrared spectroscopy (fNIRS), positron emission tomography (PET), single-positron emission computed tomography (SPECT), and magnetic resonance spectroscopy (MRS), have revealed abnormalities in brain function in patients with ADHD. fMRI is a non-invasive, high spatial resolution technology detecting functional changes in the brain through changes in cerebral blood oxygen levels. The complex pathophysiology of ADHD has been further investigated by resting-state MRI (rs-fMRI) and task-state MRI (task-MRI) functional magnetic resonance imaging studies (Plichta and Scheres, 2014; Massat et al., 2018; Cortese et al., 2021). rs-fMRI reflects the resting-state brain activity of children with ADHD. Task-fMRI can provide insight into the functional characteristics of the brain of children with ADHD during the performance of different tasks by employing various task paradigms such as motor responses (stop-signal task, go/no-go), interference suppression (Stroop, Simon, and Eriksen lateralization tasks) and transformation tasks (Hart et al., 2013; Lei et al., 2015; Hwang et al., 2019; Kolodny et al., 2020, 2022). fNIRS is a novel neuroimaging technique without any invasion and has become popular in recent years (Gossé et al., 2022). It detects neural activity by detecting oxygen and deoxyhemoglobin in the brain. fNIRS has a high tolerance for the head movements of subjects in experiments and is therefore particularly suitable for examining brain function in ADHD in a natural environment (Kohl et al., 2020). SPECT and PET are nuclear medicine techniques providing metabolic and functional information to examine the neurotransmitter dopamine in ADHD (Dubey et al., 1983; Gualtieri and Hicks, 1985). A meta-analysis study disclosed that the dopamine transporter density increased by 14% in patients with ADHD by analyzing nine studies on PET and SPECT (Fusar-Poli et al., 2012). MRS is a reliable method to detect neurotransmitters *in vivo*. Dysfunction in the metabolism of dopaminergic and glutamate has been considered to be a cause of ADHD (Perlov et al., 2009).

The application of the neuroimaging technique in ADHD has a history of more than 30 years (Zametkin et al., 1990), but the research hotspots and emerging trends have not been studied in a systematic manner. Thus, this study considered that comprehensively identifying the previous research hotspots and current research directions and defects was of high value for the subsequent research. Bibliometric analysis can analyze publications, such as books or journal articles quantitatively and qualitatively (Roldan-Valadez et al., 2019). Bibliometrics is chosen because it can analyze the contributions and cooperation of authors, institutions, countries, and journals, and judge the development and new trends of relevant literature based on current research.

To understand the current status of ADHD neuroimaging literature and identify new research directions, the software CiteSpace and Scimago Graphica were used to perform a bibliometric visualization analysis. The present study could provide a global perspective for clinical practitioners, neuroimaging experts, and researchers.

## Methods

### Retrieval method

This study was conducted by searching the Science Citation Index Expanded (SCI-Expanded) in the Web of Science (WoS). The relevant literature was published between 1 January 1992 and 20 September 2022, and the search subject terms were identified as (ADHD OR “Attention Deficit Disorder with Hyperactivity” OR “Hyperkinetic Syndrome” OR “hyperkinetic disorder” OR “Attention Deficit Hyperactivity Disorder”) AND (Neuroimaging OR “Functional Neuroimaging” OR “structural magnetic resonance imaging” OR “functional magnetic resonance imaging” OR fMRI OR “Magnetic resonance imaging” OR “Magnetic resonance^*^” OR MRS OR Tomography OR “Positron Emission Tomography” OR “Tomography, Emission-Computed” OR PET OR “Single-Photon” OR “Single Photon Emission Computed Tomography” OR PECT OR “functional near-infrared spectroscopy” OR fNIRS OR “Diffusion Tensor Imaging” OR DTI). The literature search was constrained to articles and review articles, eliminating other document types.

### Data processing

Search results were processed using Microsoft Office Excel 2019 (Microsoft, USA), and a polynomial regression model was constructed, aiming to predict the number of articles in 2022. The data from the WoS were exported in “Full Record with Cited References” format and saved with the prefix “download.” “Node Types” select “Author,” “Institution,” “country,” and “Keyword” for visual analysis to generate a visual co-occurrence map. “Pruning” selects “Pathfinder” and “Pruning sliced networks” to prune each slice of the network to reduce the density of the network and improve its readability.

**Figure 1:**
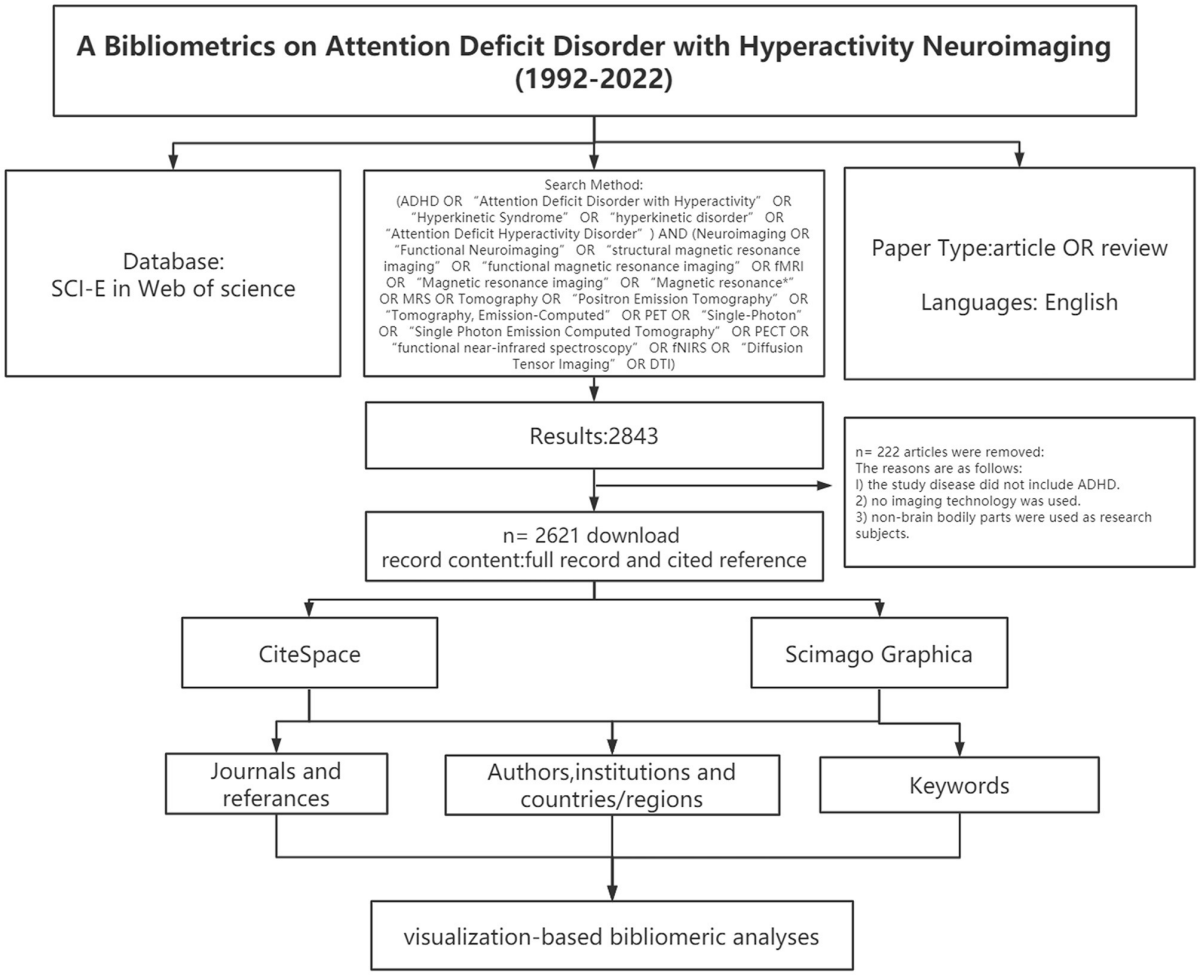
Frame flow diagram shows the detailed selection criteria and bibliometric analysis steps of ADHD neuroimaging.

CiteSpace was employed for constructing a base map for hotspots and knowledge in nephrotoxicity research, and for centrality detection (Chen and Chen, 2005). The time-slicing was performed from January 1992 to September 2022 (1 year per slice), node type (1 per time), selection criteria (g-index: *k* = 15), and pruning (pathfinder and pruning sliced network). Scimago Graphica 1.0.25 visualized the collaborative relationships of authors from different countries. In the visual diagram, each node was deemed as an element, and its size referred to the frequency of occurrence. Cooperation or connection among various elements was marked with a connection line. The node with high betweenness centrality (>0.1) is the key point in this field, which is also a concern of CiteSpace. The purple ring represents centrality. Highly cited elements are often considered to be the research hotspots in this field. The flow diagram of this study is shown in Figure 1.

## Result

### Analysis of publications a year

**Figure 2:**
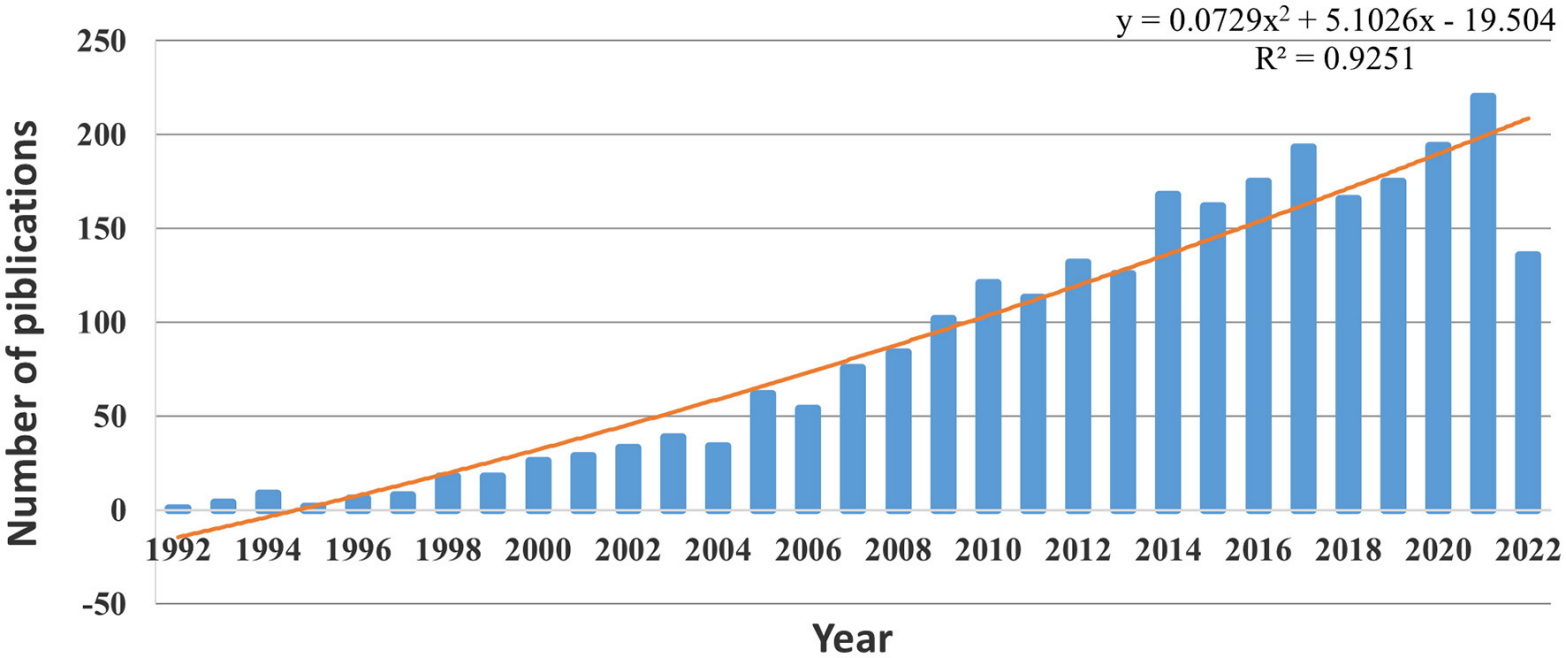
Trends in the number of publications on ADHD neuroimaging from 1992 to 2022.

A total of 2621 articles were included. Figure 2 lists the number of publications broken down by year, illustrating an overall increase from 1992 to 2022. Since 2009, the number of publications has exceeded 100 each year. After fitting these data, the results showed that the statistical relationship between the year and the number of publications was *R*^2^ = 0.9251, indicating a significant correlation. Based on the fitting curve, this study predicts that the number of relevant articles on ADHD neuroimaging will reach 210 in 2022.

### Analysis of countries/regions

**Figure 3:**
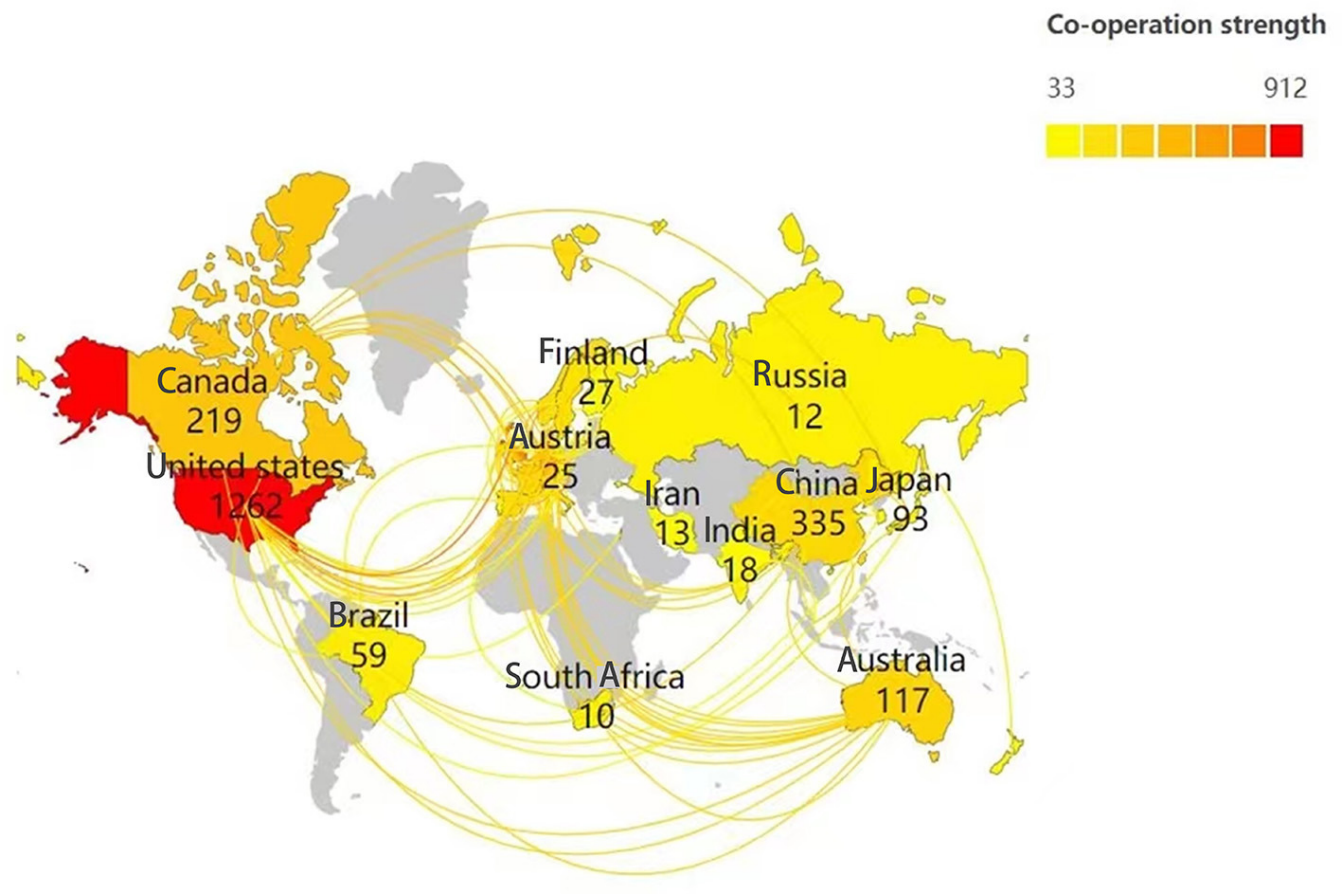
Collaboration network of countries researching ADHD neuroimaging.

Scimago Graphica was used to analyze the cooperation of authors from various countries. As shown in Figure 3, retrieved articles on ADHD neuroimaging were from 65 countries including Asia, Africa, Europe, North America, and South America (with the greatest contribution).

**Figure 4:**
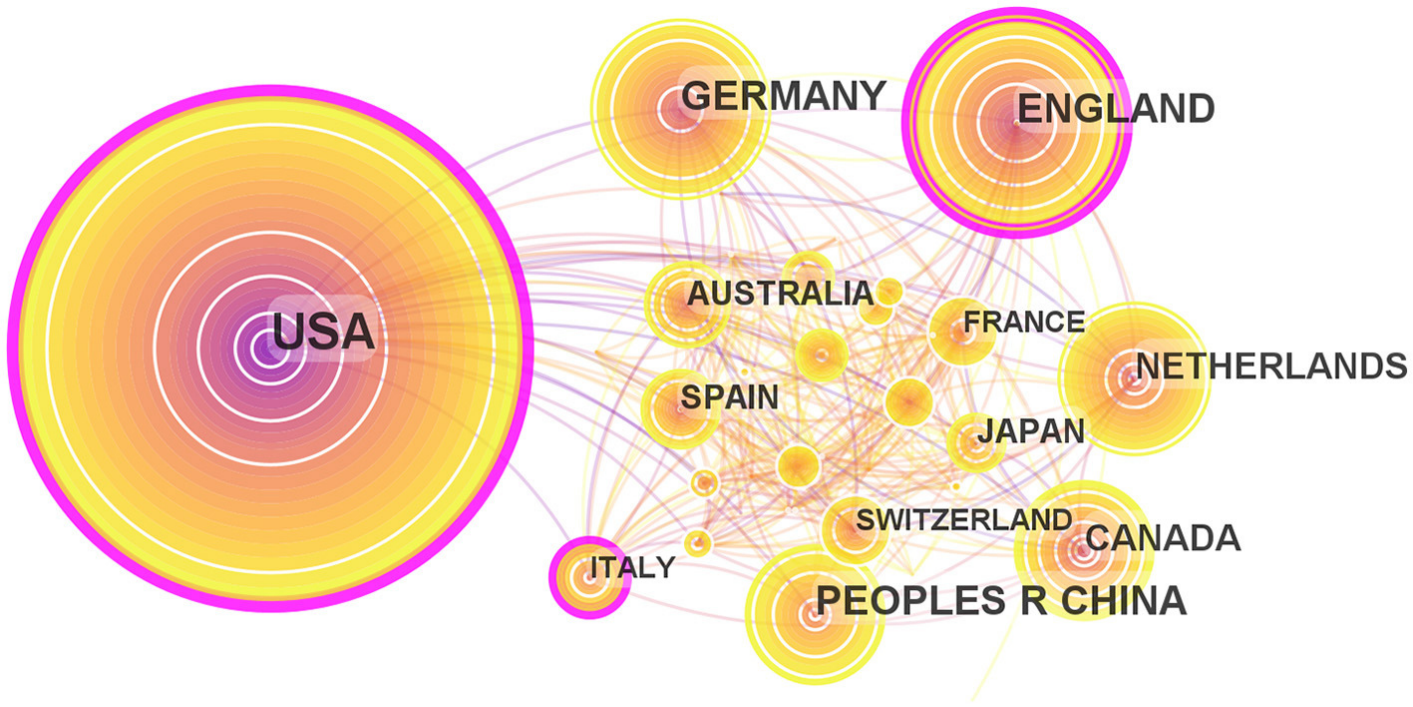
Map of countries researching ADHD neuroimaging from 1992 to 2022.

Maps drawn after the national visual analysis include 65 countries/regions and 326 links. As shown in Figure 4, these countries have greater centrality (>0.1) (purple rings). The top three countries with the greatest centrality are the United States (0.28), England (0.17), and Italy (0.16). These three countries are influential in the field of ADHD neuroimaging. The United States (*n* = 1,262, 48.15%), England (*n* = 371, 14.15%), and Germany (*n* = 326, 12.43%) published the most articles. The United States contributed almost half of all publications, with the first article published in 1993 which was earlier than any other country, showing great contribution in volume and importance. Table 1 quantifies the main findings. The top five countries (except the United States) published their first articles on ADHD neuroimaging around 1998.

**Table 1:** Top five countries with publications on ADHD neuroimaging from 1992 to 2022.

**Rank**	**Countries**	**Count**	**Centrality**	**Year**
1	USA	1,262	0.28	1993
2	England	371	0.17	1998
3	Germany	326	0.05	1998
4	China	278	0.01	2001
5	Netherlands	244	0.05	1997

### Analysis of institutions

**Figure 5:**
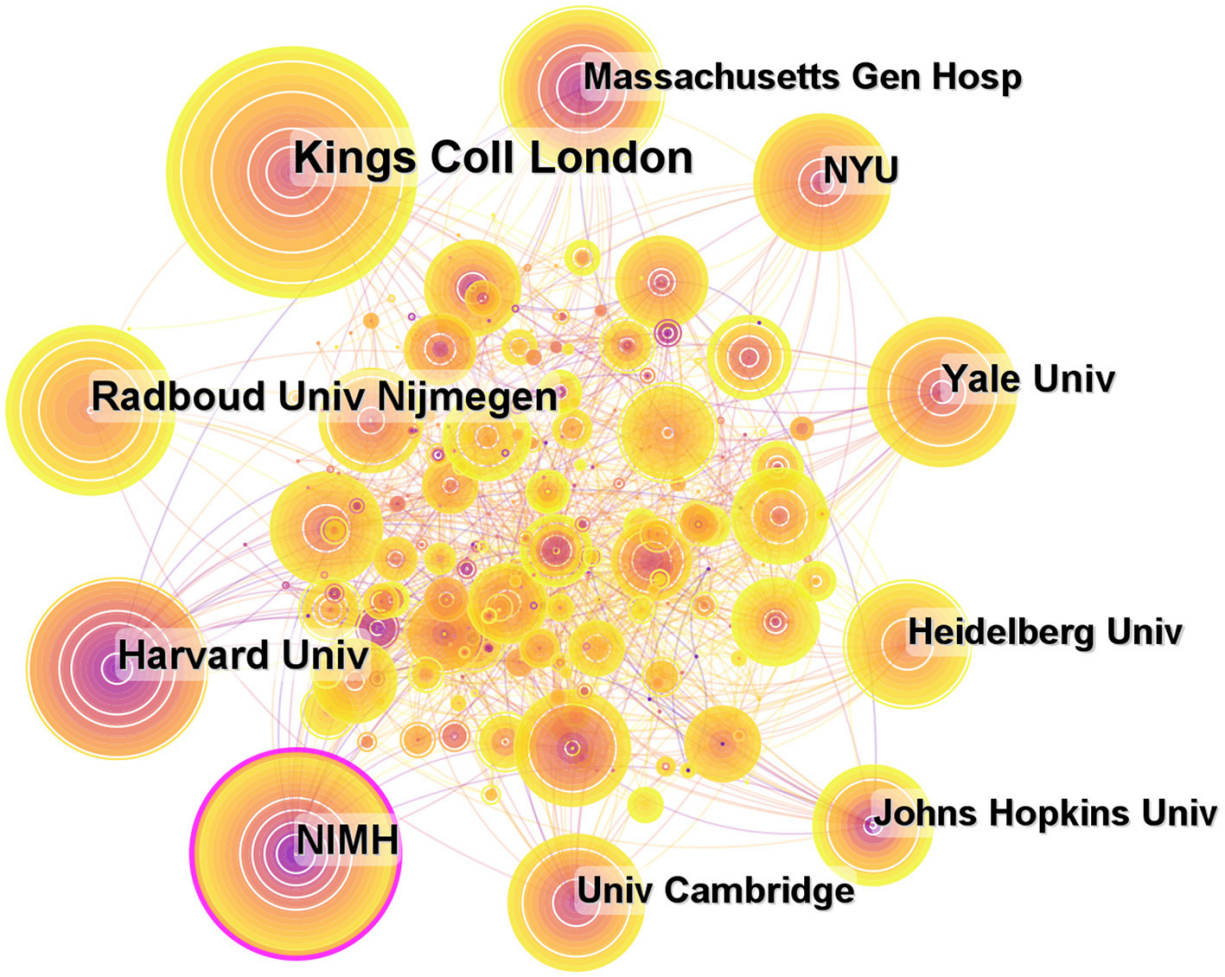
Map of institutions researching ADHD neuroimaging from 1992 to 2022.

A total of 435 institutions contributed to publications on ADHD neuroimaging (Figure 5). The top 10 institutions contributed 875 articles (33.38%). Five American institutions are represented among the top 10 institutions: Kings College London (*n* = 168, 19.2%), Harvard University (*n* = 103, 11.8%), Radboud univ Nijmegen (*n* = 101, 11.5%), National Institute of Mental Health (*n* = 96, 10.8%), and Yale University (*n* = 72, 8.2%). It is worth noting that all these institutions are located in developed countries. The National Institute of Mental Health (NIMH) has a greater centrality (0.18). NIMH published the first article in 1993 about the child and adolescent psychopathology research (Table 2).

**Table 2:** Top 10 institutions with publications on ADHD neuroimaging from 1992 to 2022.

**Rank**	**Institutions**	**Country**	**Count**	**Centrality**
1	Kings coll London	England	168	0.06
2	Harvard Univ	USA	103	0.02
3	Radboud univ Nijmegen	Netherlands	101	0.04
4	NIMH	USA	96	0.18
5	Yale Univ	USA	72	0.08
6	Massachusetts Gen Hosp	USA	71	0.03
7	NYU	USA	69	0.05
8	Heidelberg Univ	Germany	68	0.05
9	Univ Cambridge	England	66	0.03
10	Univ Groningen	Netherlands	61	0.05

### Authors and co-cited authors

**Figure 6:**
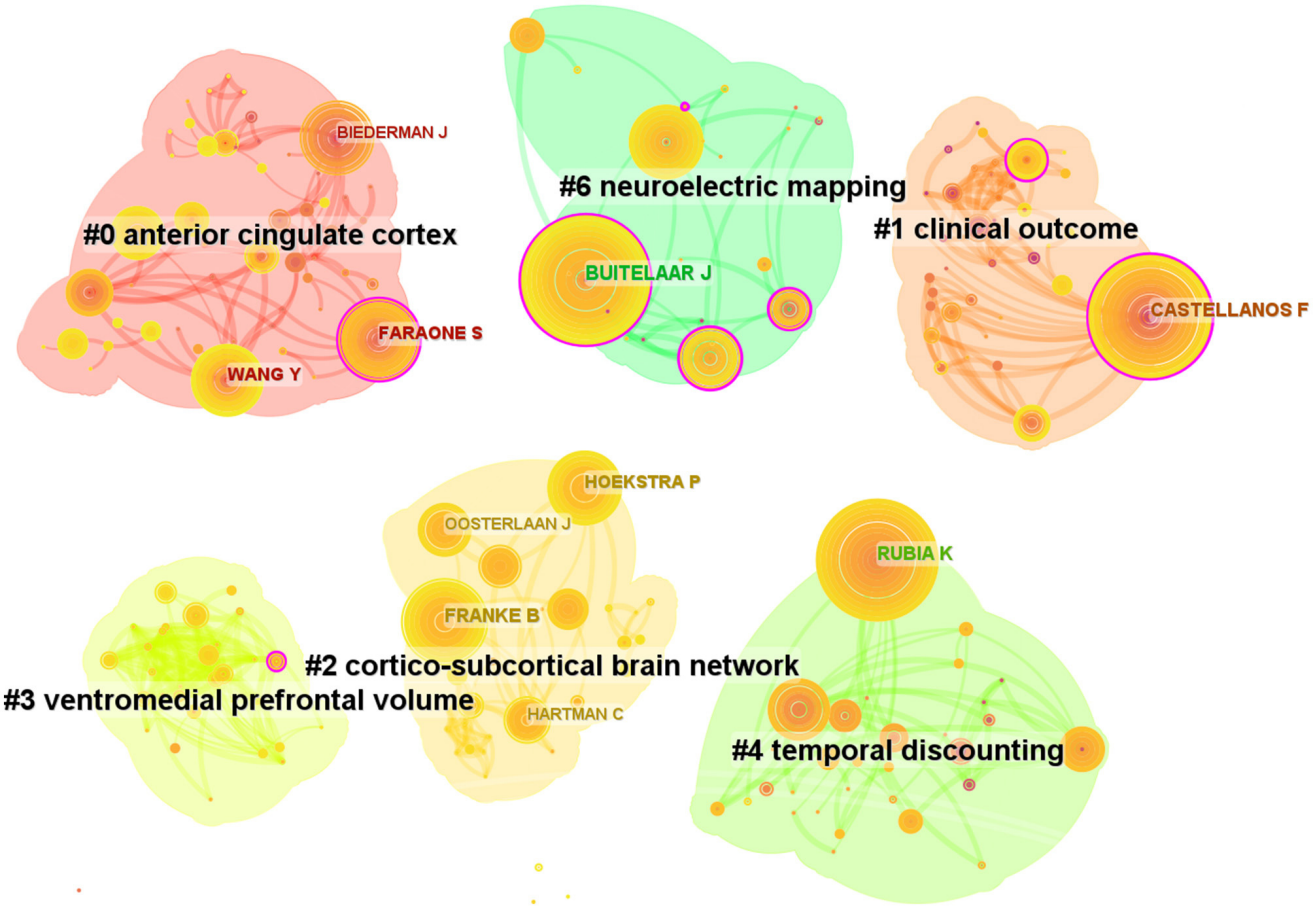
Map of authors related to ADHD neuroimaging from 1992 to 2022.

After analyzing the authors of these 2,621 articles, we obtained 634 nodes, 1,097 links, and six clusters (Figure 6). Among these authors, the top five authors were Buitelaar, J.K. (*n* = 86, 3%), Rubia, Katya (*n* = 69, 2.6%), Franke, Barbara (*n* = 62, 2.3%), Castellanos, Francisco Xavier (*n* = 61, 2.3%), and Hoekstra, Pieter J (*n* = 46, 1.7%; Table 3). Buitelaar, J.K. from Radboud University Nijmegen has the largest number of published papers, with an h-index of 112. Meanwhile, Buitelaar, J.K. has a greater centrality (0.19). His main research areas are Psychiatry, Neurosciences Neurology, and Psychology. He has published the most papers in collaboration with Franke, Barbara, who ranks third in the number of papers, and he is also from Radboud University Nijmegen. Their first collaboration could be backdated to 2011. From the cluster summary, the authors focused on the anterior cingulate cortex, clinical outcome, cortico–subcortical brain network, ventromedial prefrontal volume, temporal discounting, and neuroelectric mapping. The map of the authors' country is shown in Tables 3, 4. Castellanos FX (*n* = 922) had the highest citation counts, followed by Rubia K (*n* = 775), Barkley RA (*n* = 581), Shaw P (*n* = 532), and Biederman J (*n* = 480; Table 4).

**Table 3:** Top 10 frequency and centrality of authors related to ADHD neuroimaging.

**Rank**	**Frequency**	**Author**	**Country**	**Centrality**	**h-index**
1	86	Buitelaar J	Netherlands	0.19	30
2	69	Rubia K	England	0.03	54
3	62	Franke B	Netherlands	0.05	26
4	61	Castellanos FX	USA	0.13	46
5	46	Hoekstra P	Netherlands	0.08	20
6	44	Faraone S	USA	0.16	33
7	43	Wang Y	China	0.04	27
8	37	Oosterlaan J	Netherlands	0.06	23
9	36	Biederman J	USA	0.08	34
10	35	Hartman C	Netherlands	0.01	20

**Table 4:** Top 10 frequency cited authors related to ADHD neuroimaging.

**Rank**	**Frequency**	**Author**	**Country**	**centrality**	**h-index**
1	922	Castellanos FX	USA	0.09	46
2	775	Rubia K	England	0.06	54
3	581	Barkley RA	USA	0.09	45
4	532	Shaw P	USA	0.03	23
5	480	Biederman J	USA	0.04	34
6	469	Bush G	USA	0.02	15
7	442	Faraone SV	USA	0.06	32
8	441	Durston S	Netherlands	0.04	27
9	432	Volkow ND	USA	0.07	27
10	388	Sonuga-barke EJS	England	0.03	12

The h-index value is based on a list of publications ranked in descending order by the Times Cited count. The h-index is based on the Web of Science Core Collection citations of the publications shown on this author's record on ADHD neuroimaging.

### Analysis of journals and cited journals

Journals publishing the most studies on ADHD neuroimaging are listed in Table 5 and are in the field of psychiatry and imaging. Biological Psychiatry published the most articles on ADHD neuroimaging, and the American Academy of Child and Adolescent Psychiatry occupied the highest impact factor.

**Table 5:** Top five most productive journals related to ADHD neuroimaging.

**Rank**	**Publications**	**Journal**	**Countries**	**IF**	**CiteScore**
1	107	Biological Psychiatry	USA	12.81	21.50
2	98	Neuroimage	USA	7.40	11.20
3	88	Psychiatry Research Neuroimaging	Netherlands	2.49	4.10
4	78	Journal of the American Academy of Child and Adolescent Psychiatry	Netherlands	13.11	13.30
5	75	Human Brain Mapping	USA	5.40	8.40

IF, impact factor; IF in the category according to Journal Citation Reports (2021).

**Figure 7:**
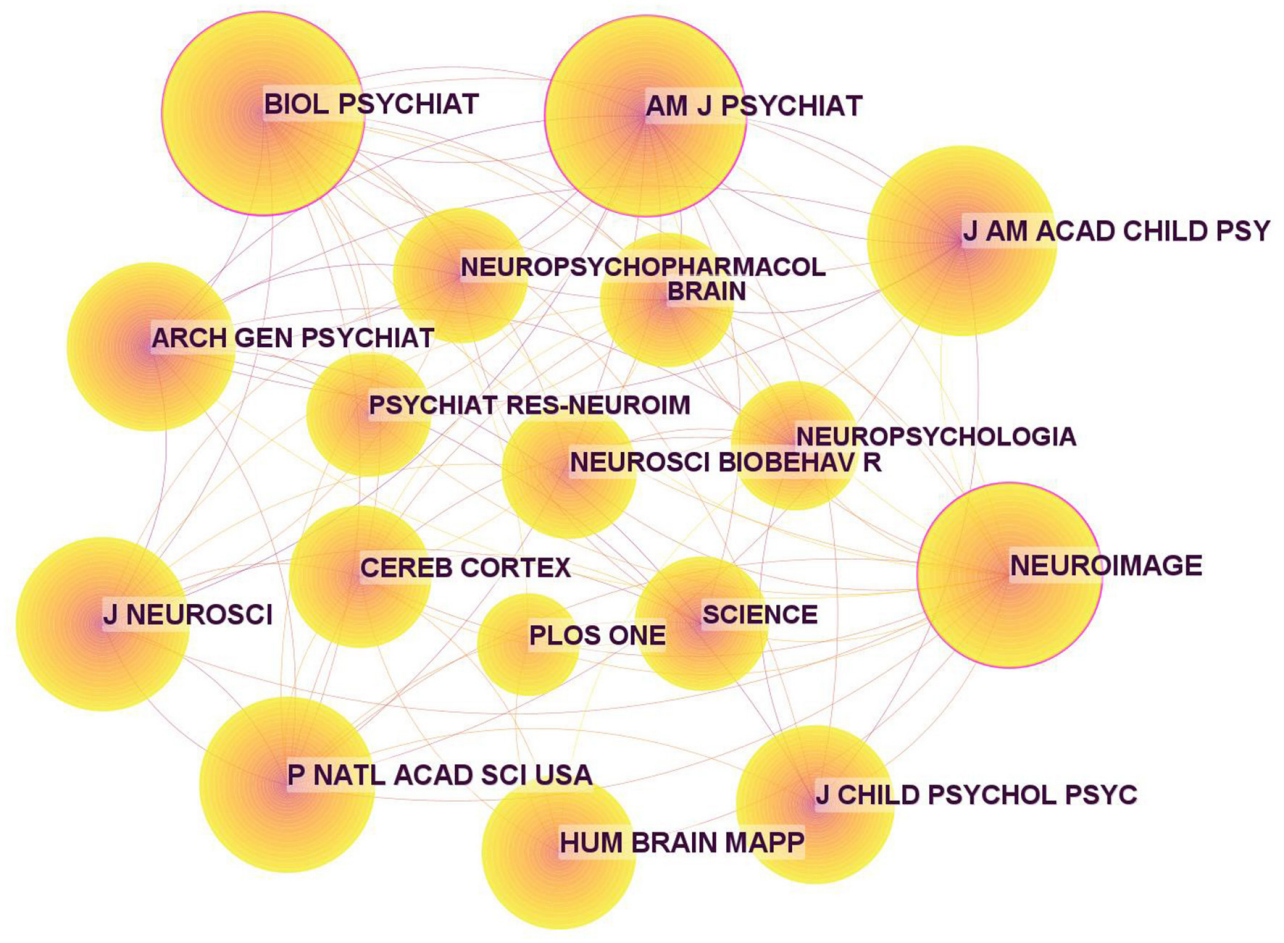
Map of cited journals for ADHD neuroimaging from 1992 to 2022.

In addition, the journals on ADHD neuroimaging cited in these publications are shown in Table 6, and the corresponding map based on CiteSpace (Figure 7) generated 1,000 nodes and 5,948 links. Three nodes were marked with purple rings, indicating high centrality. The highest was 0.13 from Biological Psychiatry. The second highest centrality was Neuroimage with a centrality of 0.12. These two journals have a large number of publications and citations and play a pivotal role in ADHD neuroimaging.

**Table 6:** Top 10 cited journals related to ADHD neuroimaging.

**Rank**	**Frequency**	**Cited journal**	**Centrality**	**Countries**	**IF**	**CiteScore**
1	2,070	Biological Psychiatry	0.13	USA	12.81	21.50
2	1,999	Neuroimage	0.12	USA	7.40	11.20
3	1,863	American Journal Psychiatry	0.10	USA	19.242	20.4
4	1,629	American Academy of Child and Adolescent Psychiatry	0.04	Netherlands	13.113	13.30
5	1,518	Proceedings of the National Academy of Sciences of the United States of America	0.06	USA	12.779	16.2
6	1,476	Journal of Neuroscience	0.03	Netherlands	6.709	5.20
7	1,430	Human Brain Mapping	0.08	USA	5.40	8.40
8	1,260	Archives of General Psychiatry	0.07	USA	14.48	N/A
9	1,211	Journal of Child Psychology and Psychiatry	0.07	England	8.265	12
10	1,176	Cerebral Cortex	0.02	USA	4.861	9.1

IF, impact factor; IF in the category according to Journal Citation Reports (2021).

### Analysis of keywords

We used the frequency of certain keywords significantly increasing or even reaching the outbreak state within a given period of time as an indicator to analyze the new trend of cutting-edge topic activity (Chen and Chen, 2005). Based on the presence of these keywords, we generated an overall network graph (802 nodes and 4,229 links) to assess the current hottest topics. There were 8 keywords that occurred more than 200 times (Table 7).

**Table 7:** Occurrences of top 15 keywords.

**Rank**	**Keyword**	**Frequency**	**Centrality**
1	Children	782	0.06
2	Prefrontal cortex	380	0.02
3	Respond inhibition	355	0.05
4	Functional connectivity	288	0.01
5	Brain	271	0.08
6	Working memory	270	0.01
7	Adolescent	264	0.02
8	Executive function	223	0.03
9	fMRI	196	0.03
10	Anterior cingulate cortex	177	0.03
11	Methylphenidate	168	0.06
12	Adult	167	0.05
13	Basal ganglia	153	0.05
14	MRI	147	0.09
15	Cognitive control	139	0.02

**Figure 8:**
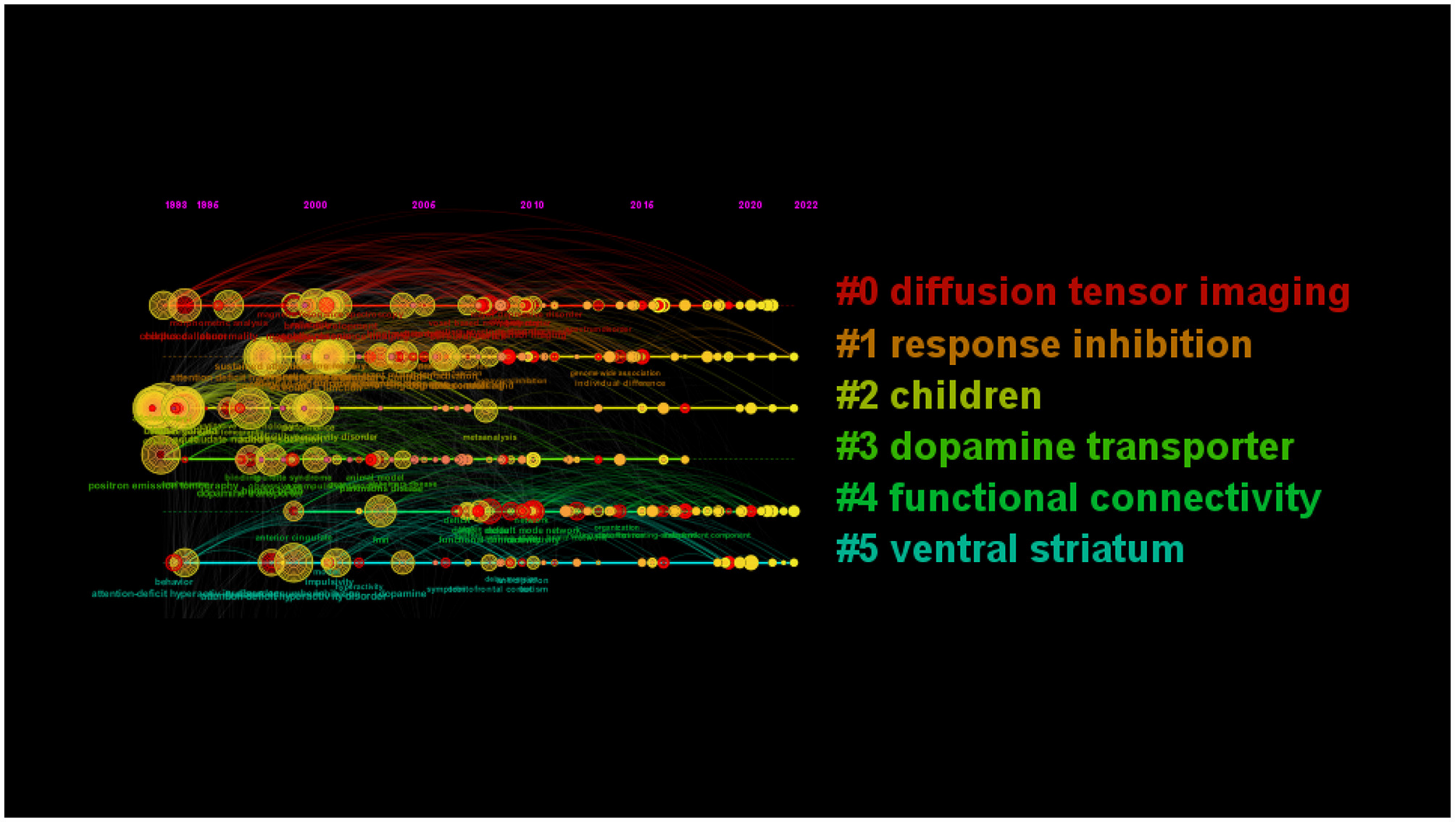
Clustering map and timeline of keywords related to ADHD neuroimaging.

Nine clusters reflected topical trends, including “white matter,” “response inhibition,” “children,” “positron emission tomography,” “functional connectivity,” and “ventral striatum.” In the timeline view, keywords from one cluster are in the same horizontal line. The longer the time span is, the longer the cluster lasts, representing the rise, prosperity, and decline of research in different periods. The timeline view in Figure 8 shows the six clusters. Cluster#0 included “corpus callosum,” “white matter,” “voxel-based morphometry,” and so on; cluster#1 included “inhibitory control,” “prefrontal cortex,” “stroop task,” and so on; cluster#2 included “childhood onset,” “boy,” “child psychopathology,” and so on; cluster#4 included “resting state network,” “deep learning,” “default network,” and so on.

**Figure 9:**
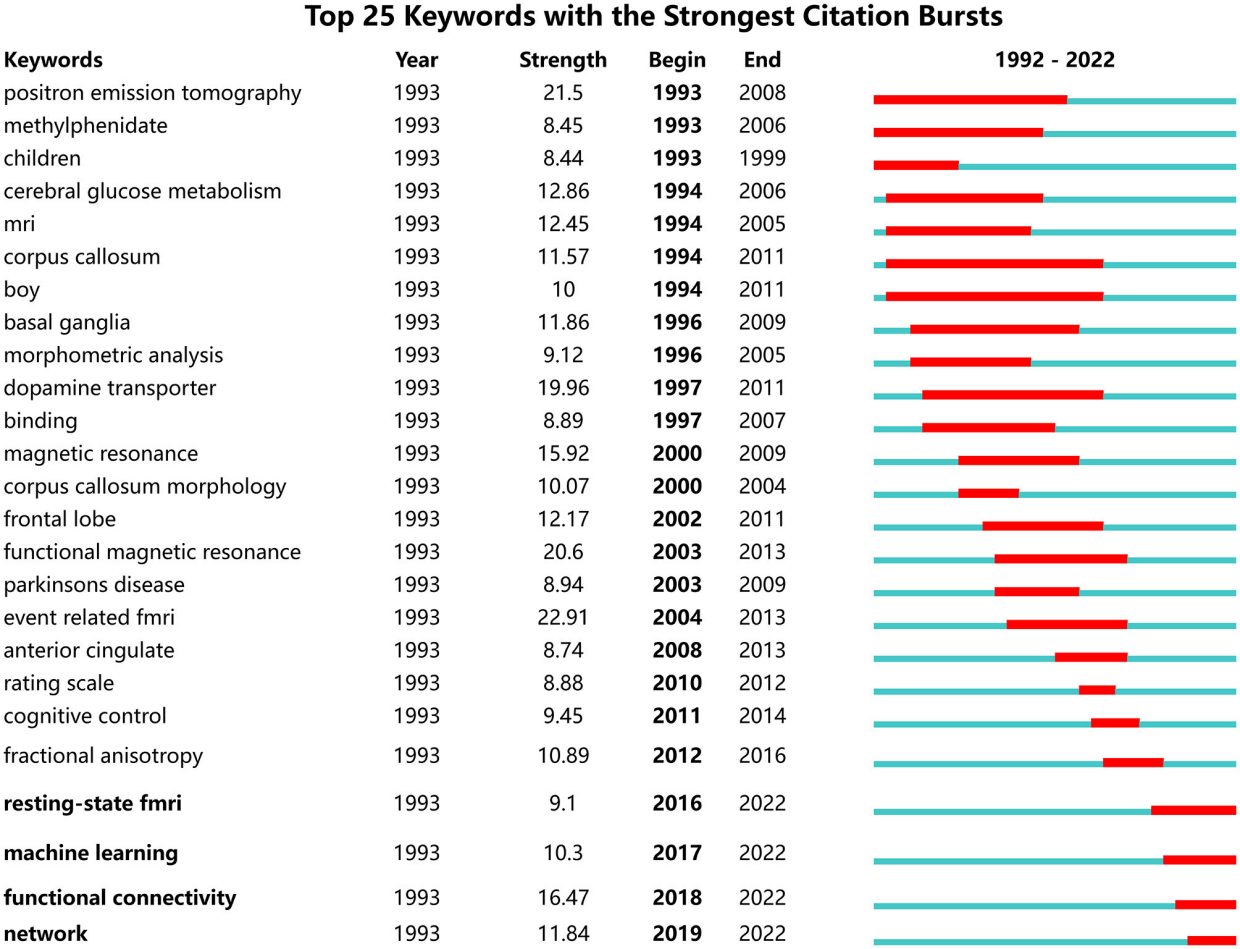
Top 15 keywords with the strongest citation burst.

As shown in Figure 9, the green and red lines refer to 1992–2022 and the periods of each keyword, respectively. “Strength” indicates the emergence intensity of keywords. The ones with high outbreak intensity are: “event related fmri” (2004–2013, strength 22.84), “positron emission tomography” (1993–2008, strength 21.13), “functional magnetic resonance” (2003–2013, strength 20.55), “dopamine transporter” (1997–2011, strength 18.58), and “magnetic resonance” (2000–2009, strength 15.22).

### Analysis of references

**Figure 10:**
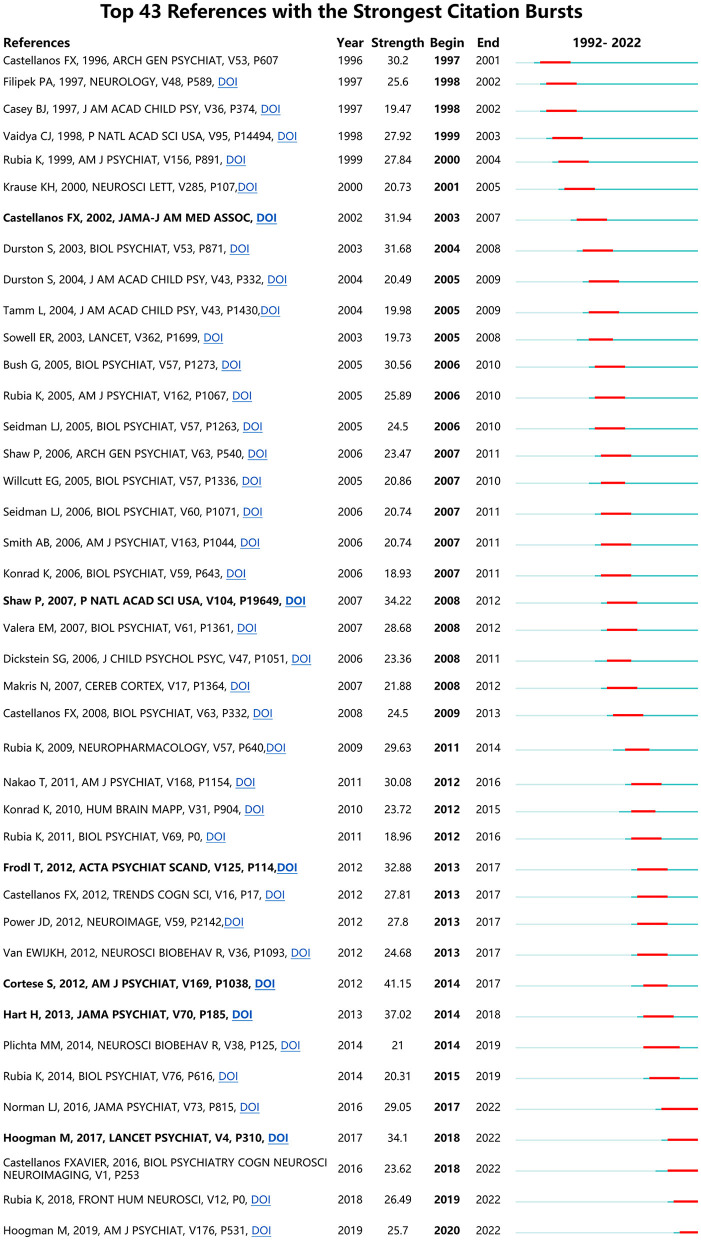
References with the strongest citation burst on ADHD neuroimaging.

If a reference has the strongest citation bursts (CBs), it is deemed as a knowledge basis of the research frontiers (Fitzpatrick, 2005; Synnestvedt et al., 2005). Figure 10 shows ADHD neuroimaging references with the strongest CB from 1992 to 2022. CBs by the end of 2022 were led by Cortese et al. (2012) (41.15), followed by Shaw et al. (2007); Frodl and Skokauskas (2012); Hart et al. (2013); Hoogman et al. (2017), and Castellanos et al. (2002).

## Discussion

The current study applied a visualized bibliometric method for analyzing the contributions and cooperations of authors, institutions, countries, journals, and emerging trends of neuroimaging in ADHD. From 1992 to 2022, the number of documents has shown a steady upward trend. A total of 2621 publications were collected from SCI-Expanded. All papers were published by 435 institutions from 65 countries/regions in 549 peer-reviewed journals.

In terms of the literature published by the country, the United States ranks first in both the number and centrality of literature, suggesting that the country plays a leading role, which is inextricably linked to the priority that the United States places on ADHD research. The National Institutes of Health (NIH)'s brain program, which was launched in the United States in 2013, has significantly increased funding and promoted ADHD neuroimaging research. The American periodical Biological Psychiatry (IF = 12.81) also has the most articles and citations. It is noteworthy that China, which ranks fourth in terms of the number of papers issued, is the only developing country in the top five. Research of the United Kingdom, Germany, and the Netherlands possess a central and significant position. Currently, most studies are conducted in developed countries such as Europe and the United States. Some scholars proposed that further research from less developed countries is needed to understand the interactions of neurobiological and socio-cultural elements in ADHD (Cortese and Coghill, 2018).

The number of papers published by King's College London ranked first accounting for 6.4% of the total number of studies. The Institute of Psychiatry, Psychology & Neuroscience (IoPPN) at King's College London is the premier for neurosciences research in Europe. Its cited publications about mental health were the most (the first 1%) (SciVal 2019). However, an analysis of these institutions showed that articles on ADHD were mostly published by research universities, including a small number of hospitals. ADHD will evolve toward precise clinical phenotypes in the next few years. Thus, relevant research units should establish cooperation with hospitals with accurate diagnostic capabilities as increasing clinicians publish literature on ADHD.

In this study, we learn more about the outstanding authors who contribute greatly to studies in this field. Author information can clearly identify the author's contribution to the field and establish potential partnerships. Buitelaar J, Rubia K, and Franke B contribute the greatest number of publications in the field of ADHD neuroimaging. Buitelaar J comes from the University of Bergen, and his research interests include ADHD, autism, and aggressiveness and impulsivity problems. Dr. Buitelaar takes an integrative strategy that includes clinical and phenotypic research, neuroimaging, cognitive research, genetics, pharmacology, and preclinical techniques. Rubia, K is a professor at King's College London, and his expertise is the pharmacological imaging of ADHD drugs and neurotransmitter manipulations, PET imaging in adults with ADHD, and so on. Franke B comes from Radboud University Nijmegen Medical Center. Dr. Franke B studies the influences of genetic factors on mental health problems and traits such as ADHD and focuses on understanding the biological mechanisms explaining the effects of such factors on the etiology of mental problems. In addition, we found that these three authors were members of the World Federation of ADHD and the ENIGMA-AHDH Working Group. Buitelaar J is the vice president of the World Federation of ADHD, while Franke B is a co-founder of ENIGMA. Through these organizations, researchers can strengthen communications and cooperation between different countries or institutions in this field. Scientific researchers including these three authors published the “The World Federation of ADHD International Consumption Statement” in 2021. Through an in-depth study of more than 2,000 participants, the authors came up with 208 specific descriptions of ADHD, which pointed out seven pieces of evidence-based evidence about the brain difference of ADHD (Faraone et al., 2021).

Keywords of “Children” and “prefrontal lobe” have the highest frequency and high centrality, which indicates that they are hot topics in research. ADHD is common in children whose brains are still developing, allowing scientists to conduct long-term longitudinal research on the brain structure of the whole life cycle. The prefrontal lobe is responsible for planning and organizing, as well as attention control, executive function, and other advanced processes. The prefrontal growth retardation in patients with ADHD leads to brain failure to effectively control behavioral activities, attention, and emotion. The development of fNIRS also promotes the research of the prefrontal lobe (Gossé et al., 2022).

In addition, the recent burst keywords are rs-fMRI, machine learning, functional connectivity, and network.

(1) The rs-fMRI can be used to investigate spontaneous brain neural activity during the rest state without requiring the preformation of any task (Anwar et al., 2016; Qian et al., 2018). Recently, different rs-fMRI computing approaches have been created based on functional segregation integration. ADHD has featured a lack of behavioral regulation, inattention, impulsivity, and hyperactivity. It might be difficult for participants with ADHD to complete task-fMRI scanning due to the symptom of hyperactivity. However, rs-fMRI does not require tasks, making it easier for participants with ADHD to comply and reducing the chance of head movement abnormalities. Thus, the rs-fMRI has been widely used in the mechanistic and clinical investigation of ADHD.
(2) Machine learning: at present, guardian interview and clinical observation are mainly used to determine whether a child suffers from ADHD, which may vary greatly because of regional divergence. If a single scale or indicator is used to diagnose or predict ADHD, the reliability will be insufficient. Therefore, we suggest that some data (such as sociodemographic information and neuroimaging) can be used to assist except the scale and relevant indicators. Increasing evidence shows that machine learning (ML) can be applied to neuroimaging data for the diagnosis and efficacy prediction of ADHD (Li et al., 2019). The largest ADHD-related dataset adopted by ML-based classifiers is ADHD-200 Global Competition, which includes 776 ADHD-afflicted youngsters ages 7–21. The results also showed that few studies focused on the classifiers for adults with ADHD and that most studies were carried out with small sample sizes (<100 patients) based on the datasets (McLeod et al., 2014; Uddin et al., 2017). In addition, due to the presence of several psychopathologies, it is more challenging to diagnose adults with ADHD than individuals in childhood. Neuroimaging should be used in the future to identify brain biomarkers in ADHD adults, objectively quantify brain structure and function, and develop and implement effective machine learning methods that will aid in objectively diagnosing, monitoring treatment responses, and predicting results (Uddin et al., 2017).
(3) Functional connectivity: ADHD is considered a neurodevelopmental disease with “brain disconnection,” including structural and functional connectivity disorder. Functional connectivity (FC) analyses are a common method of analyzing fMRI data (McLeod et al., 2014). rsFC technology has been proven to be able to measure the correlation of brain activity in the resting brain region. Thus, scholars have commonly used it to analyze the abnormalities and interaction of brain functional networks in ADHD. Dynamic functional connectivity (DFC) is an extension of the traditional resting-state functional connectivity analysis, which refers to the phenomenon that the observed functional connectivity changes in a short time (Fateh et al., 2022). At present, DFC is associated with a variety of neurological diseases and is considered to be an accurate representation of the brain's functional network. The study found that children with subtypes of ADHD exhibited obvious and broad aberrations in static FC and DFC among the resting-state networks, forming the cortical and subcortical areas compared to typically developing children (Shaw et al., 2007). Another study concluded that the etiology of ADHD could be better interpreted by the changes in brain FC (Hoogman et al., 2019). However, there are few articles on DFC related to ADHD.
(4) Network: In recent years, there has been increasing acceptance that ADHD is a distributed functional network disorder. Patients with ADHD suffer from different degrees of alternation in default mode network (DMN), frontoparietal network (FPN), ventral attention network (VAN), dorsal attention network (DAN), affective network (AN), somatosensory network (SSN), and visual network (Rubia, 2018). However, results vary in different research. Some literature found that DMN showed weaker FC in patients with ADHD than that in healthy controls (Fair et al., 2010; Choi et al., 2013; Sripada et al., 2014), while other literature provided almost opposite results (McCarthy et al., 2013; Barber et al., 2015). Comparing these results can be difficult due to differences in analytic approaches and variances in nomenclature and functional network boundaries. A meta-analysis found that the abnormal activity of fronto–striato–cerebeller networks in patients with ADHD was related to neuropsychological impairment (Hart et al., 2013). The multilayer network is a new type of network model that is increasingly being employed in neuroscience to portray the dynamic interaction between different functional systems.

References with the strongest CB published recently may help us to investigate the research hotspots in this field. Listed in Figure 8, (1) Cortese et al. (2012) discussed the task-based functional MRI studies of ADHD using a meta-analysis, which included 55 studies (39 in children and 16 in adults). This study found that children with ADHD showed significant hyperactivation within the default and ventilatory networks compared with typically developing children. In adults, ADHD mainly is caused by deficits in visual, dorsal attention, and default networks. This article provided evidence of cognitive, sensorimotor, and other neurological disorders in patients with ADHD. At the same time, the study found that the early model of ADHD was concerned about the prefrontal–striatal circuits. (2) Hart et al. (2013) conducted a meta-analysis to analyze the fMRI images of participants with ADHD, aiming to explore the relationship between age and long-term stimulant medication use effect. The study found that participants with ADHD exhibited weaker ability in cognization in distinct fronto-basal ganglia-thalamic networks when they were required to complete some inhibition and attention-related actions. (3) Shaw et al. (2007) used computational neuroanatomic techniques to estimate the cortex thickness from 824 MRI images of 223 children with ADHD and 223 controls. This study found that ADHD was exhibited as a delayed cortical maturation. Furthermore, this delay was especially noticeable in prefrontal areas that controlled cognitive functions such as attention and motor planning.

The latest valuable cited literature provides valuable information for future research directions. Hoogman et al. (2019) used extensive research to pinpoint brain traits associated with ADHD and found that children with ADHD had widespread minor abnormalities in cortical surface area. Despite the presence of ADHD diagnosis, future longitudinal research should clarify individual lifespan trajectories that result in non-significant findings in adolescent and adult groups. Rubia (2018) focused on the fMRI-based cognitive neuroscience of ADHD and recent clinically relevant applications such as fMRI-based diagnostic classification or neuromodulation therapies, aiming to correct fMRI deficits with neurofeedback (NF) or brain stimulation.

## Limitation

The current research is subjected to some limitations. First, only articles from the WoS and English articles were included, which might lead to bias in language and publication. Second, some studies unpublished or published newly were not included in this study. Third, the number of citations and centrality of the articles might vary depending on the particular time when the search was conducted. However, it is certainly believed that it has clarified the overall condition, emerging trends, and future directions of studies on ADHD, showing high reference values and providing some valuable suggestions for further study.

## Conclusion

In conclusion, this study revealed the status of relevant studies, hot topics, and trends concerning neuroimaging in ADHD from 1992 to 2022. During the past three decades, the number of relevant articles has increased significantly, which suggests that neuroimaging in ADHD has a positive development prospect. Most ADHD neuroimaging articles are from developed countries, and the authors are encouraged to develop close cooperation with others from various institutions and countries. Notably, “resting-state Fmri,” “machine learning,” “functional connectivity,” and “network” may be the hot topics in this field. With the rapid development and maturation of brain imaging technology, scholars could conduct more relevant research about the diagnosis and prognosis of ADHD.

## Data availability statement

The original contributions presented in the study are included in the article/supplementary material, further inquiries can be directed to the corresponding author.

## Author contributions

JW and NS contributed to conceiving, designing, and revising the manuscript. HD and ZH contributed to the analysis and manuscript writing. ZL, LC, and YH contributed to data collection. JW and YZ contributed to manuscript revision. All authors contributed to the article and approved the submitted version.
